# Gain‐of‐function enhancers optimize CAR‐NK cell‐based anti‐cancer immunotherapy

**DOI:** 10.1111/imcb.70113

**Published:** 2026-04-10

**Authors:** Emma Wong, Fernando Souza‐Fonseca‐Guimaraes

**Affiliations:** ^1^ Frazer Institute, Faculty of Health, Medicine and Behavioural Sciences The University of Queensland Woolloongabba QLD Australia

## Abstract

Schematic overview of the two‐stage screening approach used to identify NK cell fitness genes. (A) CRISPRa mechanism, showing dCas9‐VP64‐mediated upregulation of target genes. (B) Whole‐genome CRISPRa screening in HER2‐CAR‐NK92 cells transduced with a CRISPR sgRNA library and transferred into mice bearing HT29 tumours, followed by tumour collection and next‐generation sequencing (NGS). (C) Barcoded ORF mini‐screen in primary peripheral blood NK (PBNK) cells transduced with HER2‐CAR and an ORF library, transferred into HT29 tumour‐bearing mice, with subsequent tumour collection and NGS analysis.

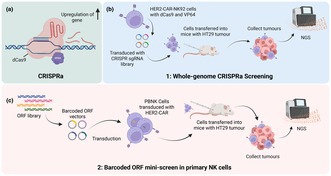

Chimeric Antigen Receptor (CAR) Natural Killer (NK) cells have powerful potential as a cell‐based immunotherapy against cancer, having the ability to target specific antigens on cancer cells.^1^ However, CAR‐NK cells can face several limitations in solid tumors, such as limited tumor infiltration and persistence in the tumor microenvironment (TME). Gene discovery techniques have been employed to identify genes which can be edited to optimize CAR‐NK cells and address some of their limitations. A recent study by Yang and colleagues in Nature performed a gain‐of‐function (GOF) discovery to identify genes which enhance NK cell function and incorporate these into CAR‐based engineering as a “booster.”^2^ The gene encoding olfactory receptor family 7 subfamily A member 10 (*OR7A10*), a G protein‐coupled receptor, was identified as a key enhancer of CAR‐NK cell effector functioning. Engineering CAR‐NK cells with *OR7A10* cDNA substantially enhanced NK tumor‐killing functions *in vitro* and *in vivo*. Their findings highlight the potential of this approach as a scalable strategy to improve CAR‐NK cell therapeutics and address the limitations currently facing the field. Furthermore, Yang and colleagues' study exemplifies the power of employing GOF identification of genes that optimize NK cells and immunotherapies.

Recently, cell engineering approaches to introduce further detection/activation signals such as CAR in NK cells have shown powerful potential to precisely enhance their tumoricidal functions in cancer patients. Clinical trials of allogeneic CAR‐NK cells have shown favorable outcomes in the treatment of hematological malignancies.^3^, ^4^ Furthermore, CAR‐NK cells show promise against unfavorable side effects, commonly associated with CAR‐T cells, for example, minimal risk of graft‐versus‐host disease (GvHD) and cytokine release syndrome following allogeneic transplantation.^4^, ^5^

Although CAR‐NK‐based therapy holds promise, several strategies are being investigated to explore their potential against solid cancers, such as knockout of inhibitory regulators and cytokine engineering.^1^ Furthermore, a novel and promising strategy involves incorporating gain‐of‐function genetic “boosters” into their engineering, unlike previously reported gene knockout strategies. Clustered regularly interspaced short palindromic repeats (CRISPR) technologies have revolutionized research in cellular therapies. Its ability to guide and recognize specific DNA sequences allows a multitude of applications and has attracted great interest for optimizing cellular immunotherapies. Although it is routinely used for genomic manipulation by precise gene editing, CRISPR can be utilized for high‐throughput screening and gene discovery in cells. This can be useful in determining genetic factors that underlie important processes in immune responses against diseases such as cancer. One such technique is called CRISPR activation (CRISPRa), involving incorporation of inactive CRISPR‐associated (Cas) protein engineered with transcriptional activators [such as four tandem copies of the Herpes Simplex Viral Protein 16 (VP64)]. In this methodology, the single‐guide RNA (sgRNA) can be designed to target and activate specific gene promoters, thereby boosting their expression and enabling functional studies of those genes.^6^ CRISPRa mechanism is illustrated in Figure 1a.

**Figure 1:**
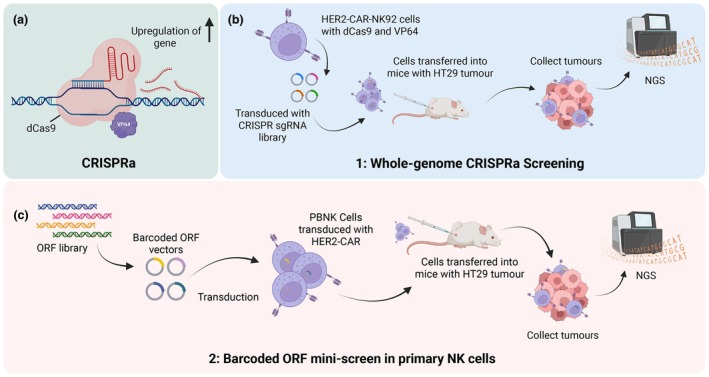
Schematic Overview of Identification of Genes that Enhance the in vivo Anti‐tumor Efficacy of CAR‐NK Cells. **(a)** Generation of HER2‐CAR‐NK cells using NK92 cells which were further transduced (lentivirus) with dCas9‐VP64‐Blast and MS2‐P65‐HSF1‐Hygro, followed by lentiviral transduction with the CRISPR sgRNA library. The cells were then adoptively transferred to NOD‐scid Il2rg‐null (NSG) mice which had been inoculated with human colorectal adenocarcinoma cell line, HT29‐GFP–luciferase cells. The tumors were then collected and underwent Next Generation Sequencing (NGS) to determine the top gene targets, establishing which single‐guide RNAs (sgRNAs) were enriched or depleted. This was to achieve initial screening and hit prioritization. The sgRNAs which were more abundant indicate corresponding “enhancer” genes. **(b)** Mechanism of Clustered Regularly Interspaced Short Palindromic Repeats activation (CRISPRa), whereby the inactive dCas9 fused to transcriptional activator four tandem copies of the Herpes Simplex Viral Protein 16 (VP64) causes upregulation of the genes guided to by the sgRNA. **(c)** Primary human CAR‐NK barcoded open reading frame unique molecular identifier (ORF UMI) mini‐screen approach used to validate top hits. The barcoded ORF library was pool‐cloned into expression vectors designed to drive overexpression of each candidate gene using Gibson Assembly. Using lentivirus vectors, each barcoded ORF was transduced into HER2‐CAR‐PBNK cells, which were then transferred into HT29 tumor‐bearing NSG mice. The tumors were then extracted and subjected to NGS to analyze the barcode representations. Figure made in ©BioRender – biorender.com.

Yang and colleagues recently explored this strategy in a Nature 2026 study, implementing a technique that utilized CRISPR‐based *in vivo* screening in NK cells to identify genes whose overexpression enhances anti‐tumor function.^2^ This *in vivo*, genome‐scale approach facilitated unbiased discovery of genes that regulate CAR‐NK cell functionality within the TME. This method was paired with an *in vivo* barcoded open reading frame (ORF) screen which focuses on validation of the top hits to further analyze their anti‐tumor activity. Yang *et al*. used these techniques, performing an *in vivo* CRISPRa screen to identify GOF targets that enhance CAR‐NK efficacy (Figure 1b), followed by a targeted ORF screen to validate top hits (Figure 1c). This approach identified and confirmed the gene Olfactory Receptor Family 7 Subfamily A Member 10 (*OR7A10*), a G protein‐coupled receptor, as a key enhancer, or “booster,” in a workflow as illustrated in Figure 1.^2^

OR7A10 was further tested for enhanced efficiency in NK cells, both within *in vitro* tumor environments and by culturing. This was achieved by generation of OR7A10 overexpression (OE) construct, which was packaged into lentivirus vectors and used to transduce peripheral blood‐derived primary NK cells (PBNK) from healthy donors (Figure 2a). Results indicated that when OR7A10 was engineered into CAR‐NK cells, there was increased NK cell proliferation and persistence as shown by higher expansion and count at the end of the culture. Furthermore, OR7A10(OE)‐transduced CAR‐PBNK cells elicited upregulation of the chemokine receptor C‐X‐C motif chemokine receptor 2 (CXCR2) (required for cell trafficking to tumor sites), as well as increased activation markers such as Cluster of Differentiation 69 (CD69), Interleukin‐2 receptor alpha chain (CD25), and co‐stimulatory receptor Tumor Necrosis Factor Receptor Superfamily Member 9 (4‐1BB) when stimulated with human colon adenocarcinoma (HT29) cells. Using a repeated challenge assay, OR7A10(OE)‐transduced CAR‐PBNK cells also showed reduced exhaustion markers, T‐cell immunoglobulin and mucin‐domain containing‐3 (TIM‐3), Lymphocyte‐activation gene 3 (LAG‐3), Programmed cell death protein 1 (PD‐1), and Natural Killer Group 2 member A (NKG2A) when co‐cultured with HT29 cells.

**Figure 2:**
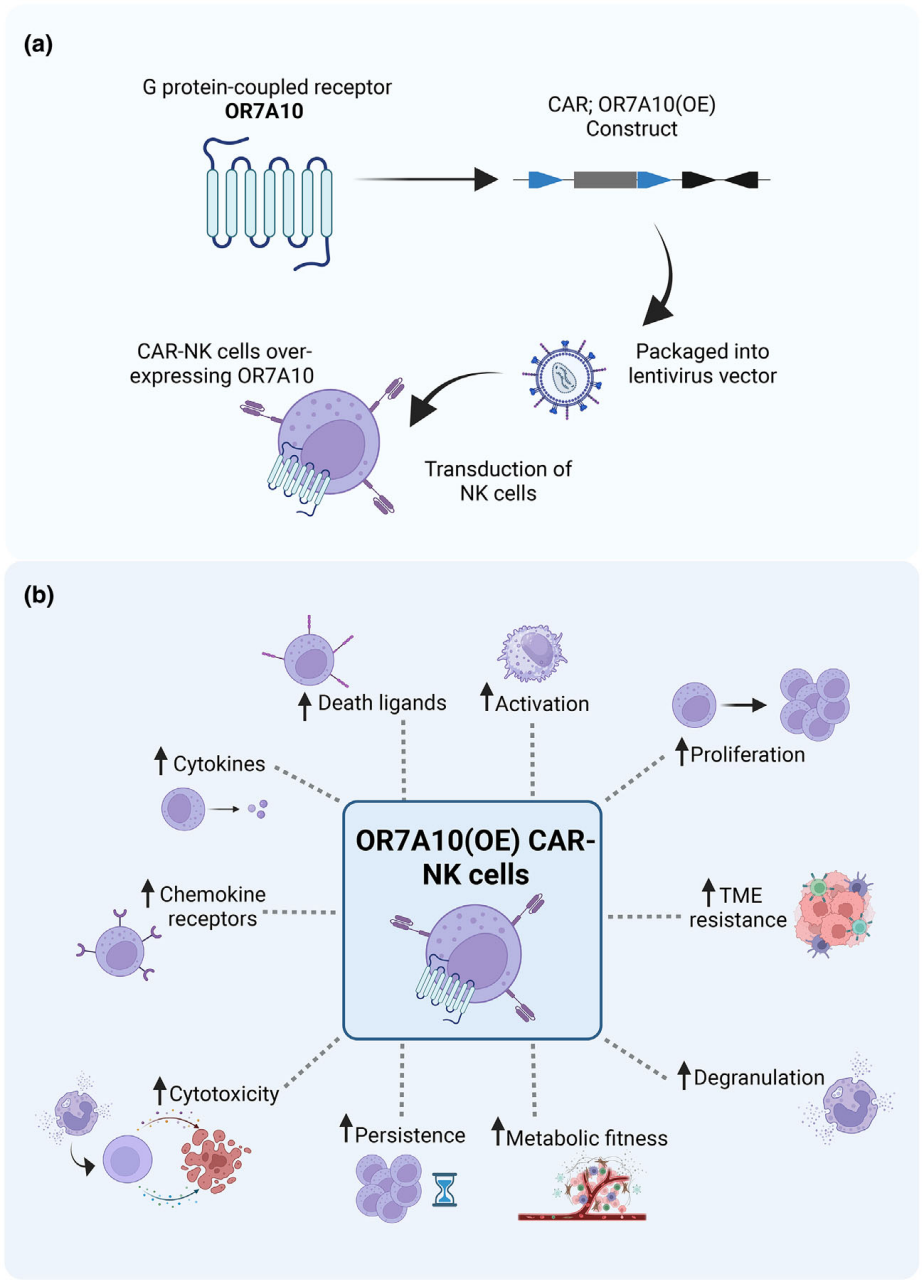
Schematic Illustrating OR7A10 Overexpression in CAR‐NK Cells and Consequent Functional Enhancements. **(a)** Generation of CAR/OR7A10 overexpression (OE) construct, which is packaged into lentivirus vectors and used to transduce NK cells. The result is a CAR‐NK cell overexpressing OR7A10 receptor via cDNA overexpression. **(b)** Enhanced effector functions observed in vitro against cell lines of the CAR‐NK cell overexpressing OR7A10 (OR7A10(OE) CAR‐NK cells), acting as a NK cell “booster.” Figure made in ©BioRender – biorender.com.

Furthermore, OR7A10 was able to enhance effector function of CAR‐NK cells, significantly elevating granzyme B and perforin expression and increasing their degranulation after HT29 co‐culture. Likewise, OR7A10 was able to stimulate increased death receptors [Fas‐Ligand and Tumor Necrosis Factor‐Related Apoptosis‐Inducing Ligand (TRAIL)] and increased production of interferon‐gamma (IFN‐γ) and Tumor Necrosis Factor (TNF) compared with controls. In addition, the authors tested the effect of overexpressing OR7A10 in CAR‐NK cells within the immunosuppressive conditions of the TME. In this setting, they found significantly enhanced cytotoxicity across all conditions tested, including acidity, hypoxia, adenosine signaling agonist, calcineurin inhibitors, and transforming growth factor‐beta (TGF‐β). These findings establish how this “booster” has the potential to overcome the immunosuppressive TME in solid tumors. A summary of the enhanced function of CAR‐NK cells with incorporation of OR7A10 can be seen in Figure 2b. Notably, all *in vivo* experiments were conducted in immunocompromised xenograft mouse models, and future studies using immunocompetent systems will be essential to fully recapitulate the complexity of the human TME and validate OR7A10‐driven reprogramming in the context of intact host immunity.

This landmark study has provided the field with not only the identification of a novel target that can be optimized as a booster to incorporate within CAR‐NK cells to assist in overcoming the suppressive TME, but a robust application for CRISPRa in discovering GOF genes with therapeutic potential. The evidence that OR7A10 is an efficient NK cell booster is particularly favorable as their study considered key limitations in CAR‐NK manufacturing and scalability in the study.^2^ Unlike other classically immunosuppressive targets such as (transforming growth factor‐beta receptor 2) *TGFBR2* which require genetic knockout,^7^ a functional booster can be incorporated within the same CAR construct. This approach offers a much simpler and scalable option for enhancing CAR‐NK cells. Their study integrated a HER2‐CAR/OR7A10 construct, whereby the booster was able to be successfully engineered into the CAR construct targeting HER2. When transduced into immortalized cell line, NK92 and tested *in vivo*, it showed near complete tumor eradication in NOD‐scid *Il2rg*‐null (NSG) mice with HT29 colorectal adenocarcinoma tumors. Furthermore, the clinical feasibility of incorporating OR7A10 into CAR‐NK products was evaluated using NK cells derived from cord blood (CBNK) from healthy donors. OR7A10(OE)‐transduced CAR‐CBNK cells exhibited significantly enhanced cytotoxicity against HT29 cells compared with controls. The study further optimized the CAR construct by integrating an (Interleukin‐15) IL‐15 expression design sequence, engineering a HER2‐CAR/hIL‐15/OR7A10 construct, to generate CAR‐CBNK cells. These CAR‐NK cells displayed further enhanced cytotoxicity against the same HT29 colorectal adenocarcinoma cells. Looking forward, OR7A10 could be incorporated into many CAR‐binder types depending on the type of tumor category to be treated, and the discovery of novel scFvs is also a fast‐evolving field of its own.^7^ Pre‐clinical assessment of new generations of CAR‐NK cells that incorporate gain‐of‐function boosters have a bright future for this field.

Genome‐wide CRISPR screens have also been notable in key and recent studies exploring optimization of NK cells and their application in immunotherapy. For example, CRISPR screening in NK cells elucidated insights about the IL‐15 signaling pathway, identifying the enzymes NEDD8 E2‐conjugating (UBE2F) and ubiquitin E3‐ligase (ARIH2) to be essential for activating the Cullin‐5 RING E3 (CRL5) ligase complex, which degrades the IL‐15R complex and inhibits IL‐15 signaling. Therefore, it was observed that targeting these enzymes enhances CAR‐NK cells and *in vivo* anti‐tumor activity by increasing responsiveness to IL‐15,^8^ a critical survival and cytotoxicity enhance factor for NK cells.^9^ Similarly, a parallel study used unbiased genome‐wide CRISPR screening and identified Mediator complex subunit 12 (MED12), Ariadne RBR E3 ubiquitin protein ligase 2 (ARIH2), and Cyclin‐C (CCNC), which when deleted can significantly improve NK cell anti‐tumor activity. Furthermore, dual knockout of ARIH2 and CCNC was shown to improve *in vivo* efficacy of CAR‐NK anti‐tumor efficacy.^10^ These recent studies also reinforce that CRISPR screening is a powerful methodology to discover key regulators involved in NK cells, which can be further applied to improve cellular immunotherapies.

In conclusion, the studies discussed, particularly the recent Yang *et al*. study, explore the promising opportunities of CAR‐NK optimization, primarily centered around using genome‐wide CRISPR screens to identify genes which can be targeted to enhance CAR‐NK function within the TME. Specifically, validating targets which can be incorporated into the CAR (such as OR7A10), acting as “boosters” to increase proliferation and activity, and overcoming the immunosuppressive TME.

## AUTHOR CONTRIBUTIONS

**Fernando Souza‐Fonseca‐Guimaraes:** Conceptualization; supervision; funding acquisition; writing – review and editing; writing – original draft. **Emma Wong:** Conceptualization; writing – original draft.

## CONFLICT OF INTEREST

F.S.F.G. is a Board Member of Cure Cancer Australia Foundation and a member of the Scientific Advisory Committee of ANZSA. Sanofi TSH, Microba Life Sciences, and Cartherics sponsor research in the laboratory of F.S.F.G. The authors have no commercial, proprietary, or financial interest in this study.

## Data Availability

Data sharing not applicable to this article as no datasets were generated or analysed during the current study.
